# Validation of a Scale to Measure Career Concerns Related to Perceived Environmental Challenges (the CC-PEC Scale)

**DOI:** 10.3390/bs16050636

**Published:** 2026-04-24

**Authors:** Andrea Zammitti, Angela Russo, Jenny Marcionetti, Anna Parola

**Affiliations:** 1 Department of Educational Sciences, University of Catania, 95121 Catania, Italy; andrea.zammitti@unict.it; 2 Department of Human and Social Sciences, University Mercatorum of Rome, 00186 Rome, Italy; angela.russo@unimercatorum.it; 3 Department of Education and Learning, University of Applied Sciences and Arts of Southern Switzerland, 6928 Manno, Switzerland; jenny.marcionetti@supsi.ch; 4 Department of Humanities, University of Naples Federico II, 80138 Napoli, Italy

**Keywords:** career concerns, environmental challenges, scale validation, EFA, CFA

## Abstract

Choosing a future career represents a complex developmental task, often accompanied by multiple concerns and anxieties. The Social Cognitive Career Theory and Life Design paradigm emphasize the importance of supporting individuals in managing career-related challenges. However, global stressors—such as the COVID-19 pandemic, the war in Ukraine, and increasing awareness of the climate emergency—have introduced new and multifaceted sources of uncertainty that are not adequately captured by existing instruments. This gap highlights the need for a psychometrically sound measure to assess emerging career-related concerns in the contemporary context. Accordingly, the study aimed to develop and validate the Career Concerns related to Perceived Environmental Challenges (CC-PEC Scale). Four studies were conducted. Study 1 employed exploratory factor analysis, supporting a three-factor structure (Career-related COVID-19 pandemic concern, Career-related war concern, and Career-related climate emergency concern). Study 2 confirmed this structure using confirmatory factor analysis and demonstrated measurement invariance across gender, supporting a hierarchical factorial model. Study 3 provided evidence of concurrent and discriminant validity through associations with related constructs. Study 4 offered preliminary evidence of stability and predictive validity using life satisfaction and flourishing as outcome variables. Overall, the findings support the CC-PEC Scale as a reliable and valid instrument for assessing career-related concerns linked to global environmental challenges. These results have important implications for research and career guidance interventions aimed at supporting young people’s career development in increasingly uncertain contexts.

## 1. Introduction

Choosing a future career is a complex developmental task that can be overwhelming for adolescents and young adults. In contemporary societies, this process is further complicated by multiple large-scale environmental challenges, such as pandemics, wars, and the climate crisis, that increase uncertainty about the future and may hinder career planning. Understanding how these challenges are perceived and cognitively appraised is essential for designing effective career guidance interventions in increasingly uncertain contexts.

As Nota et al. (2014) argue, two theoretical frameworks, the Social Cognitive Career Theory (SCCT; Lent et al., 1994, 2000) and the Life Design (LD; Savickas et al., 2009), are particularly relevant for understanding these processes.

The SCCT (Lent et al., 1994) offers a conceptual framework to explain how individuals develop their careers through the interaction of personal and environmental factors. Grounded in Bandura’s general Social Cognitive Theory (Bandura, 1986), SCCT underscores the interplay between cognitive-personal variables (such as self-efficacy, outcome expectations, and personal goals) and environmental factors, including personal attributes, environmental characteristics, and learning experiences, in shaping career construction (Lent et al., 2000). Opportunities, resources, barriers and affordances are subject to individual interpretation, and their impact depends on how they are perceived and managed (Vondracek et al., 2019). According to Lent (2013), individuals should proactively address potential barriers by anticipating future obstacles and identifying the type and amount of resources required to achieve their goals, as well as the strategies needed to attain them.

Interventions based on the SCCT and the Life Design (Savickas et al., 2009) emphasize individuals’ ability to actively manage career challenges. The holistic approach proposed by Life Design (Savickas et al., 2009) suggests that individuals approach career construction by considering the whole life plan, emphasizing the construction of meaningful career narratives and the development of adaptive skills to navigate the complexities of career paths. Adaptability, a key capacity highlighted by LD models, is the individual’s ability to prepare for and adapt to unexpected events (Savickas & Porfeli, 2012). This skill is becoming increasingly crucial in the ever-evolving job market. The contextual nature of LD allows for a focus on the historical and cultural situation, with its attendant barriers and affordances, within which the individual needs to adapt and flourish (Savickas, 1997). Reflection is also central, as it enables individuals to make sense of barriers within their life stories (Duarte, 2018). The counsellor’s work is to support the client in exploring and understanding contextual factors, barriers, outcome expectations, and career readiness (Stebleton, 2010).

For career practitioners drawing on SCCT and LD frameworks, it is increasingly important to consider perceived environmental barriers and to examine how these shape the future planning of adolescents and young adults. According to the SCCT, career interests are more likely to develop into goals, and goals are more likely to be realized when individuals perceive fewer barriers. Specific measurement tools assessing the perceived impact of these barriers on career concerns would allow researchers and practitioners to better identify intervention targets and examine their antecedents and consequences. Hence, building on the integration of SCCT and Life Design perspectives, this study aims to develop and validate a scale assessing the extent to which young adults perceive environmental challenges as barriers to their future career planning.

### 1.1. The Contemporary Environmental Challenges

Career planning occurs in an environment increasingly characterized by multiple and overlapping global challenges that may act as barriers to future career choices. Traditionally, three major challenges were defined: technological evolution and digitization, economic recession and labour market issues, and environmental factors (Ciobanu et al., 2022; Di Maggio et al., 2020). In recent years, however, additional global threats have emerged, increasing concerns among adolescents and young people facing career decisions. Among the most relevant challenges faced in recent years are the COVID-19 pandemic, the war in Ukraine, and the climate crisis.

The COVID-19 pandemic has caused a major crisis in the global economy and labour market, with an enormous impact on people, especially young adults. The COVID-19 crisis has created additional obstacles in the transition from school to work, such as reduced hiring opportunities and increased job instability (ILO, 2020). Several studies conducted prior to COVID-19 pandemic had already identified labour market uncertainty as a significant source of anxiety among young people, often leading to disengagement and avoidance of future planning (Iovu et al., 2018; Lewchuk, 2017). The outbreak of COVID-19 further intensified the pre-existing difficulties inherent in school-to-work transitions, making the present more precarious and the future appear increasingly uncertain (Parola, 2020). Shepherd (2023) highlighted how young people at the start of their careers experienced skills mismatch, underemployment, job loss, and reduced wages. Consistently, several studies have shown an increase in fears and concerns about the future among young people and during the pandemic. For example, Andresen et al. (2021) found that in Germany almost half of young people agreed with the statement “I am afraid of my future.” Similarly, studies conducted in different countries have documented increased anxiety, fear, and perceived economic insecurity among young adults (Parola, 2020; Margolius et al., 2020; Mahmud et al., 2021). Korkmaz and Doğanülkü (2021) found that fear of COVID-19 negatively affected vision about the future, highlighting the role of future orientation in the relationship between fear of COVID-19 and career distress. Consistently, interventions focusing on future orientation and LD approaches have been shown to mitigate the negative effects of the pandemic on young people’s career planning (Santilli et al., 2021; Zammitti et al., 2023a).

The Russo-Ukrainian war is an international conflict and is also expected to have long-term economic, social and health impacts (Sheather, 2022). The European economy is already experiencing rising energy prices and cost of living (Orhan, 2022), which may indirectly affect young people’s career opportunities. Research on the direct impact of war on career development remains limited. However, many studies highlight its strong psychological impact on young people (World Health Organization, 2022), including perceived threat, anxiety, intolerance of uncertainty (Moshagen & Hilbig, 2022; Regnoli et al., 2023, 2024a; Riad et al., 2022; Skwirczyńska et al., 2022). Barchielli et al. (2022) found an association between pandemic- and war-fears among Italian young adults. Recently, Regnoli et al. (2024b) further showed that future anxiety and intolerance of uncertainty amplify the impact of war-related distress. In contrast, the link between war and future career uncertainty remains underexplored in empirical studies, although it is theoretically highlighted as a risk factor in contemporary career choices (Ginevra et al., 2021).

Finally, the ecological crisis represents a major global challenge, which Rifkin (2019) describes as part of a broader “sixth mass extinction”. As with the COVID-19 pandemic and war, young adults are among the most vulnerable groups (Hickman, 2019; J. Wu et al., 2020). Several studies highlight the impact of climate anxiety (or eco-anxiety) on the psychosocial well-being of adolescents and young people (Pihkala, 2020; Skilling et al., 2023). In particular, Hickman et al. (2021) show that climate anxiety is associated with pessimistic views of the future and feelings that humanity is under threat. More recent studies confirm the salience of climate-related concerns across different populations and cultural contexts (Cebeci et al., 2025; Hickman, 2019, 2020). In some cases, climate-related distress may also affect daily functioning and engagement with future planning. Innocenti et al. (2025) documented cases in which eco-anxiety developed into “eco-paralysis,” characterized by emotional overload, helplessness, and reduced occupational functioning. More broadly, psychological literature emphasizes that climate change is increasingly linked to stress, anxiety, and depressive symptoms, underscoring the growing relevance of ecological concerns as a psychosocial challenge with implications for future planning and life trajectories (Innocenti, 2022). Together, these findings indicate that climate-related concerns represent an ongoing phenomenon that shapes individuals’ perceptions of their future and may influence their life and career planning.

The need for more sustainability, peace, equity and health is central to the United Nation 2030 Agenda, which represents the “future we want” (Santilli et al., 2023). Accordingly, career guidance aimed at supporting adolescents and young people should consider these emerging global challenges and help individuals navigate an increasingly complex and uncertain future. Taken together, these global challenges share key characteristics such as unpredictability, global impact, and long-term consequences, and may function as perceived barriers shaping young people’s career development and future planning. However, research has largely examined these challenges in isolation, highlighting the need for integrative approaches capable of capturing their combined psychological impact on career development.

### 1.2. The Present Study

The threats addressed, i.e., the COVID-19 pandemic, the war in Ukraine and the climate change, have in common that they undermine the labour market and make it difficult for individuals to imagine a stable and predictable future. In recent years, several psychological instruments, mostly of a clinical nature, have been developed to measure emotional responses associated with these phenomena, such as fear of COVID (Ahorsu et al., 2020; Italian validation by Soraci et al., 2020), fear of war (Kalcza-Janosi et al., 2023; Italian validation by Regnoli et al., 2023), and eco-anxiety (Hogg et al., 2021; Italian validation by Rocchi et al., 2023).

However, these instruments primarily assess domain-specific or clinically oriented emotional responses and do not capture how such global crises are cognitively appraised in relation to individuals’ career development. Thus, beyond general anxiety constructs, there is a need to assess career-specific concerns arising from the perceived impact of these events on employment opportunities and professional trajectories. To date, only the measure used by Mahmud et al. (2021) integrates these context-specific stressors within a unified framework focused on career development, but it is limited to COVID-19 and was developed only in English and Bangla, highlighting the need for additional domain-specific instruments, particularly in other cultural contexts such as Italy. Developing such an instrument would contribute to (a) advancing knowledge on how young people perceive these challenges as barriers to their career futures, (b) examining the antecedents and consequences of these perceptions, and (c) informing career guidance interventions.

Hence, building on the integration of SCCT and LD perspectives, this study aims to develop and validate an instrument to assess the extent to which young adults perceive global challenges, such as COVID-19, the war in Ukraine and climate change, as barriers to their future career choices. This approach is particularly suited to capture the multidimensional and context-dependent nature of perceived environmental barriers in contemporary career development.

Following established guidelines for scale development and validation (Beaton et al., 2000), we adopted a rigorous, multi-phase validation strategy. Four sequential studies were designed to ensure a comprehensive evaluation of the instrument. Specifically, Study 1 focused on item development and the examination of the underlying factorial structure through exploratory factor analysis (EFA). Study 2 aimed to confirm the factorial structure using confirmatory factor analysis (CFA), compare alternative measurement models, and test measurement invariance across gender. Study 3 examined convergent and discriminant validity by assessing associations with theoretically related but distinct constructs. Finally, Study 4 evaluated temporal stability through test–retest reliability and examined predictive validity with respect to indicators of well-being. This stepwise approach allowed for a systematic assessment of structural validity, measurement equivalence, construct validity, and criterion-related validity, thereby strengthening the overall psychometric robustness of the CC-PEC Scale.

## 2. Study 1: Item Development and Factor Structure of the CC-PEC Scale

The aim of the first study was to generate a pool of items assessing career-related concerns associated with the COVID-19 pandemic, the war in Ukraine, and the climate emergency, and to examine the factorial structure and reliability of the measure using exploratory factor analysis (EFA). A three-factor structure was expected.

### 2.1. Method

#### 2.1.1. Item Development

As for the first objective, five items from a previous study by Mahmud et al. (2021) were translated into Italian and used to assess future career-related concerns associated with the COVID-19. These items were selected because they specifically capture career-related cognitive concerns. Rather than generating entirely new items for each environmental challenge, we adapted the environmental referent (i.e., the war in Ukraine and the climate emergency) while keeping the career-related content constant. This approach allowed us to isolate the perceived source of concern while minimizing variance attributable to item wording. Following the guidelines proposed by Clark and Watson (1995), particular care was taken to ensure that the items were clearly worded and free from double negatives or overly complex structures. In addition, inclusive language was used to ensure gender neutrality. The final scale comprised 15 items. As recommended in the literature (Crocker & Algina, 1986), a team of six experts in the field of psychometrics and career development evaluated that the five items adequately represented the constructs under investigation. Specifically, the experts rated each item in terms of clarity and correspondence with the construct using a 4-point scale (1 = unclear and 4 = very clear or 1 = does not represent the construct and 4 = does represent the construct). Each expert also provided individual feedback for each item. No items were removed at this stage. Instructions for administration were then developed, and a five-point Likert scale ranging from 1 (Totally disagree) to 5 (Totally agree) was chosen.

#### 2.1.2. Participants and Procedure

To determine the required sample size, we employed an item-to-participant ratio of 1:10, a common rule of thumb for a priori sample size estimation in exploratory factor analysis (Costello & Osborne, 2005). Therefore, a minimum of 150 participants was required.

Convenience sampling was used, recruiting participants from the general population. Participants were required to meet the following inclusion criteria: being at least 18 years old (the age of majority in Italy), being a university student, and having sufficient proficiency in reading and understanding Italian. In order to verify the presence of these requirements, filter-questions were inserted in the demographic section of the research protocol, including the identification of age, student status (whether a university student or not) and whether individual was a native Italian speaker or not.

Participants participated in the study on a voluntary basis by completing an online survey and providing informed consent. The survey introduction described the objective of the research, the possibility of abandoning the administration at any time and the contact details of the research coordinators. Participants were also informed that the results would be presented in aggregate form, ensuring that individual responses could not be identified.

A total of 231 university students participated in the study, of whom 184 were female (79.7%) and 47 male (20.3%). The age of the participants ranged between 19 and 29 years (M = 20.22; SD = 1.42). With regard to gender, the majority of participants identified with their assigned gender, 2 participants identified as non-binary and 1 participant preferred not to answer. Only 8 participants (3.5%) had a bachelor’s degree, whereas the remaining participants had a high school diploma. Most participants were not employed; however, 21 students reported having some form of employment. All data were collected during the 2024/2025 academic year.

#### 2.1.3. Measure

In this study, we used the preliminary version of the Career Concerns related to Perceived Environmental Challenges (CC-PEC) Scale.

### 2.2. Data Analysis

First, the assumptions for conducting an EFA were verified. Specifically, we checked: (a) that the normality of the data was respected (Child, 2006). We adopted the criterion proposed by George and Mallery (2010) who consider as normal the data that show a skewness and kurtosis between −2 and +2; (b) that the sample size was adequate for an exploratory factor analysis. We used the Kaiser–Meyer–Olkin sample adequacy test (KMO) whose interpretation follows the following indications: a KMO > 0.9 is excellent, a KMO value between 0.80 and 0.90 indicates a good sample, a KMO value between 0.70 and 0.80 indicates an acceptable sample, between 0.60 and 0.70, the sample is mediocre and less than 0.60 indicates an inadequate sample (Kaiser & Rice, 1974); (c) that the correlation between the various items was significant. In this case, we used Bartlett’s test of sphericity, which provides good indications of adequacy. If the test is significant, then the matrix correlations are sufficiently high (Polit & Beck, 2020; Tabachnick & Fidell, 2019). To conduct the EFA, we used main-axis factoring with promax rotation for 15 items using SPSS 27.0 software (Grieder & Steiner, 2021). The number of factors to be extracted was determined by the number of factors with an eigenvalue greater than 1 (Horn, 1965; Polit & Beck, 2020; Tabachnick & Fidell, 2019). Prior to examining factor loadings, extraction commonalities were examined, as the literature suggests eliminating commonalities less than 0.40 (Osborne et al., 2008). Furthermore, items were considered part of a factor with a factor loading greater than 0.30 (Field, 2013).

To assess internal consistency, McDonald’s Omega index was used, whose values must be greater than 0.7 to indicate that the instrument has good reliability.

### 2.3. Results

Regarding the assumptions for conducting an EFA, results indicated that (a) skewness and kurtosis values were less than 2 in absolute value for all items, (b) Kaiser–Mayer–Olkin (KMO) test value was 0.91 and (c) Bartlett’s test was significant (χ^2^ = 2141.51; *p* < 0.000). This indicates that the data were suitable for EFA. The eigenvalue of the three factors were 7.01, 1.82 and 1.39. They accounted for 68.72% of the total variance. Minimum communality was 0.47 (item 5) and factor loadings were between 0.44 (item 5) and 0.80 (item 6). Results exposed in Table 1 show that each item loads adequately on the right factor.

McDonald’s Omega was 0.83 for Factor 1 (Career-related COVID-19 pandemic concern), 0.89 for Factor 2 (Career-related war concern), and 0.91 for Factor 3 (Career-related climate emergency concern).

## 3. Study 2: Confirmatory Factor Analysis and Gender Invariance

Study 2 aimed to test the factorial structure of the CC-PEC Scale using confirmatory factor analysis (CFA) and to examine measurement invariance across gender. We expected confirmation of the three-factor structure and evidence of gender invariance.

### 3.1. Method

#### 3.1.1. Participants and Procedure

The procedures used in this study are the same as those reported in Study 1. In this case, the sample consisted of 226 university students, including female (64.6%) and 80 male (35.4%), aged 19 to 30 years (M = 21.60; SD = 2.41). With regard to gender, most participants identified with their assigned gender; one participant identified as non-binary and one preferred not to respond. Most participants had a high school diploma (179), while the remaining participants had a bachelor’s degree (42), a master’s degree (4) or a PhD (1); 24 participants reported being employed, whereas the others were not. All data were collected during the 2024/2025 academic year.

#### 3.1.2. Measure

In this study, we used the version of the Career Concerns related to Perceived Environmental Challenges (CC-PEC) Scale as developed in Study 1.

### 3.2. Data Analysis

To test the construct validity of the scale, we used a CFA, using the Mplus software, version 8. We tested four different models: a single-factor model (Model 1), a first-order model with correlated factors (Model 2), a second-order model (hierarchical) (Model 3), a bi-factorial model (hierarchical) (Model 4). More specifically, in the single-factor model, all items loaded onto a single common factor. In the first-order model, each item loaded onto its respective latent dimension, capturing distinct facets of career-related environmental concerns. In the second-order model, items loaded onto their corresponding first-order factors, which in turn loaded onto a higher-order general factor. In the bifactor model, each item loaded simultaneously onto its specific latent factor and onto a general overarching factor. Given the response scale, the robust maximum likelihood (MLR) estimator was used.

In order to check the adequacy of the models, the following indices were taken into account: the Satorra–Bentler Chi-square test (SB χ^2^; Satorra & Bentler, 2001), Comparative Fit Index (CFI; Bentler, 1990), Tucker–Lewis index (TLI; Browne & Cudeck, 1992), Root Mean Square Error of Approximation (RMSEA; Steiger, 1990), and standardized root mean square residual (SRMR; Hu & Bentler, 1999). The following reference values were considered when reading the results: Ratio between χ^2^ and degrees of freedom less than 5 (Wheaton et al., 1977), CFI and TLI of 0.90 or higher (Bentler, 1990, 1998; Browne & Cudeck, 1992), RMSEA acceptable if less than 0.10, and SRMR less than 0.08 (Hu & Bentler, 1999). Finally, to compare the two models, we used the Akaike Information Criterion (AIC; Burnham & Anderson, 2004); lower values of AIC indicate a better model (Hair et al., 1998).

To identify the best-fitting factor structure, the following indices were computed: DIFFTEST, ΔCFI, and ΔRMSEA. The following cutoffs were used to detect the best factorial solution: DIFFTEST equal to Δχ^2^ with *p* value > 0.050, ΔCFI < 0.01, and ΔRMSEA < 0.015 (Chen, 2007). To identify the best-fitting factor structure, model fit indices were compared across alternative models; following Chen (2007), a model was considered inferior when at least two of the three recommended cut-off criteria were violated. Finally, Akaike information criterion (AIC) and Sample size adjusted Bayesian Information Criterion (BIC) were also used. Models with the lowest AIC and BIC values are considered to show the best fit to the data.

To further examine the dimensionality of the scale and the extent to which a general factor accounts for the shared variance among items, additional indices were computed. Specifically, the Explained Common Variance (ECV) and Omega hierarchical (ωH) were calculated based on the standardized loadings of the model. ECV was used to estimate the proportion of common variance attributable to the general factor, while ωH was employed to assess the proportion of reliable variance explained by the general factor relative to the total score (Rodriguez et al., 2016). These indices provide complementary information for evaluating whether the construct is better represented as unidimensional or multidimensional within a hierarchical framework.

To assess invariance across gender, a multiple-group confirmatory factor analysis (CFA) was conducted. The analysis was conducted on the best-fitting model. The sample was divided into two groups based on gender. We assessed invariance across three levels of measurement invariance: configural, metric, and scalar invariance. Measurement invariance was evaluated using DIFFTEST, ΔCFI, and ΔRMSEA. The following cutoffs were used to detect the best factorial solution: DIFFTEST equal to Δχ^2^ with *p* value > 0.050, ΔCFI < 0.01, and ΔRMSEA < 0.015 (Chen, 2007). To identify the best-fitting factor structure, model fit indices were compared across alternative models; following Chen (2007), a model was considered inferior when at least two of the three recommended cut-off criteria were violated.

### 3.3. Results

A series of confirmatory factor analyses (CFAs) were conducted to examine the factorial structure of career-related environmental concerns (Table 2). The single factor model demonstrated poor fit to the data, χ^2^(90) = 818.40, CFI = 0.608, RMSEA = 0.189, indicating that a single-factor solution was inadequate. The first-order correlated model demonstrated substantially improved fit, χ^2^(87) = 212.62, CFI = 0.932, RMSEA = 0.080. Similarly, the second-order model showed comparable fit indices, χ^2^(88) = 214.01, CFI = 0.932, RMSEA = 0.080. The bifactor model also demonstrated acceptable fit, χ^2^(75) = 198.32, CFI = 0.934, RMSEA = 0.085.

Chi-square difference tests and changes in incremental fit indices were examined to determine the best-fitting factorial structure (Table 3). The unidimensional model showed a significantly worse fit compared to the first-order model, Δχ^2^(3) = 605.78, *p* < 0.001, exceeding the recommended cutoffs for ΔCFI and ΔRMSEA. The second-order model did not significantly worsen model fit relative to the first-order solution, Δχ^2^(1) = 1.40, *p* = 0.237, with ΔCFI = 0.000 and ΔRMSEA = 0.000, indicating factorial equivalence. Similarly, the bifactor model did not significantly improve model fit compared to the first-order structure, Δχ^2^(12) = 14.30, *p* = 0.282, with negligible changes in CFI and RMSEA. Considering parsimony and information criteria, the second-order model showed the lowest BIC value and comparable fit indices, supporting the hierarchical conceptualization of the construct.

Additional bifactor indices indicated that the general factor accounted for approximately 65% of the common variance (ECV ≈ 0.65), with Omega hierarchical (ωH ≈ 0.59), suggesting a multidimensional structure with a substantial general component.

Measurement invariance across gender was tested using a sequence of increasingly constrained models (configural, metric, and scalar invariance) estimated with robust maximum likelihood (MLR) (Table 4).

The configural model demonstrated acceptable fit, χ^2^(174) = 361.29, CFI = 0.906, RMSEA = 0.098, SRMR = 0.062. Constraining factor loadings to equality (metric invariance) did not significantly worsen model fit, Δχ^2^(12) = 12.89, *p* = 0.377, with negligible changes in incremental fit indices (ΔCFI = −0.002; ΔRMSEA = −0.003; ΔSRMR = 0.005). Similarly, imposing equality constraints on item intercepts (scalar invariance) did not significantly reduce model fit compared to the metric model, Δχ^2^(12) = 8.38, *p* = 0.755, with changes in fit indices well below recommended cutoffs (ΔCFI = 0.001; ΔRMSEA = −0.003; ΔSRMR = 0.002).

## 4. Study 3: Concurrent and Discriminant Validity of the Scale

To assess the validity of the newly developed scale, we conducted a comprehensive evaluation of both concurrent and discriminant validity. These were examined by correlating the CC-PEC Scale scores with established measures of fear of COVID-19 pandemic, fear of war, and environmental concern. The aim was to verify that the new constructs are related yet distinct from fear and concerns related to these environmental challenges. Accordingly, we expected low to moderate correlations (i.e., *r* > 0.10 and <0.49) between career-related concerns and fear measures referring to the same environmental challenges, as well as greater unique variance within each construct than shared variance between constructs.

### 4.1. Method

#### 4.1.1. Participants and Procedure

The minimum sample size for this study was determined a priori using the G*power 3.1.9.7 software (Faul et al., 2007), specifically applying the test statistic “correlation: bivariate normal model”. With an alpha error level set at 0.05 and a target power of 95%, assuming a correlation coefficient (ρ) under the alternative hypothesis (H1) of 0.20, the calculated minimum sample size was 266. Data were collected following the same procedure described in Studies 1 and 2. The sample comprised 271 participants, aged 19 to 30 years (M = 22.76; SD = 2.52). The gender distribution was predominantly female, with 230 participants (84.9%), followed by 39 males (14.4%), and 2 non-binary individuals (0.7%). Regarding educational attainment, the majority of participants held a high school diploma (161 participants, 59.4%). Additionally, 66 participants (24.4%) had a bachelor’s degree, 38 participants (14.0%) had a master’s degree, and 6 participants (2.2%) had a doctoral degree or post-graduate specialization. All data were collected during the 2024/2025 academic year.

#### 4.1.2. Measure

In addition to the CC-PEC Scale, the following measures were used:

Fear of COVID-19. To assess fear of COVID-19, we used three items originally developed by P. Wu et al. (2009) to measure fear of SARS, and subsequently adapted to the COVID-19 context in Italy by Zammitti et al. (2021). Participants responded to statements on a 5-point Likert scale ranging from 1 (not at all) to 5 (very much). The items were: “Thinking about COVID-19 makes me feel anxious,” “I feel tense when I think about the threat of COVID-19,” and “I feel quite anxious about the possibility of another outbreak of COVID-19.” In P. Wu et al.’s (2009) study, the scale demonstrated a Cronbach’s alpha of 0.70, while Zammitti et al. (2021) reported a Cronbach’s alpha of 0.93. Cronbach’s alpha and McDonald’s Omega values for this study were 90 and 0.90, respectively.

Fear of War Scale (FOWARS; Kalcza-Janosi et al., 2023; Italian validation by Regnoli et al., 2023). The fear of becoming a victim of war was measured using the Italian version of the FOWARS, which is divided into two dimensions: the “Physiological Dimension of Fear” (items 1–7, excluding item 3) and the “Experiential Dimension of Fear” (items 8–13). Cronbach’s alpha and McDonald’s Omega values for the scale were 0.83 and 0.83 for Physiological Dimension of Fear and 0.86 and 0.87 for Experiential Dimension of Fear, respectively.

Environmental Concern Scale (ECS; Weigel & Weigel, 1978; Italian validation by Zammitti et al., 2023b). The eight-item version of the ECS was used to assess environmental concerns, focusing on two aspects: biospheric concerns (related to animals, plants, marine life, birds) and egoistic concerns (related to oneself, lifestyle, health, and future). Participants rated their concerns on a 7-point scale from 1 (not important) to 7 (most important). Cronbach’s alpha and McDonald’s Omega values were 0.93 and 0.93 for biospheric concern and 0.85 and 0.85 for egoistic concern, respectively.

### 4.2. Data Analysis

Concurrent validity was examined by correlating the CC-PEC Scale scores with measures of fear of the COVID-19 pandemic, fear of war, and environmental concern using Pearson’s *r*. The strength of association was interpreted as follows: *r* between 0.10 and 0.29 indicated a low association, *r* between 0.30 and 0.49 indicated a moderate association, and *r* > 0.50 indicated a high association.

To evaluate discriminant validity, the Fornell and Larcker (1981) criterion was applied. First, we calculated factor loadings using CFA with Maximum Likelihood estimator. Next, we computed the Average Variance Extracted (AVE) to measure how well a latent construct explains its observed variables, with an acceptable threshold of 0.50 (Hair et al., 2010). High and significant correlations among different scales may indicate multicollinearity (Farrell, 2010; Fornell & Larcker, 1981). Discriminant validity is supported when the unique variance of each construct (AVE) is greater than the shared variance between constructs (squared factor correlation; Fornell & Larcker, 1981).

### 4.3. Results

Table 5 presents the Pearson’s correlation coefficients among the CC-PEC Scale values and the other measured variables. CC-PEC specific to COVID-19 pandemic showed a high positive correlation with CC-PEC related to war and CC-PEC related to climate emergency. CC-PEC related to war exhibited a high positive correlation with CC-PEC related to climate emergency and low associations with physiological fear of war and experiential fear of war. CC-PEC related to climate emergency was highly correlated with both CC-PEC related to COVID-19 pandemic, CC-PEC related to war, and with environmental biospheric concerns; no significant association was found with environmental egoistic concerns.

Discriminant validity for CC-PEC Scale was assessed by calculating the Average Variance Extracted (AVE) and comparing it with the squared correlation between the constructs (R^2^). As shown in Table 4, the AVE values for the CC-PEC scale were higher than the squared correlations with all the examined variables, supporting the discriminant validity of the measure (see Table 6).

## 5. Study 4: Test–Retest and Predictive Validity

Study 4 aimed to examine the predictive validity and temporal stability of the CC-PEC Scale using a two-wave longitudinal design with a 3-week interval. To assess the temporal stability of the scale, the scale was administered to the same participants at two different time points. To evaluate predictive validity, we employed a regression model to assess the predictive value of the CC-PEC Scale on life satisfaction and flourishing.

Regarding the temporal stability of the CC-PEC, high values related to the interclass correlation coefficient were expected. Regarding predictive validity, it was expected that the dimensions assessed by the CC-PEC would negatively predict flourishing and life satisfaction.

### 5.1. Method

#### 5.1.1. Participants and Procedure

The sample consisted of 34 university students, aged 21 to 42 years (M = 24.94; SD = 4.89). Thirty-two participants identified as female and two as male. Thirty-three individuals held a bachelor’s degree, and one had a master’s degree. Twenty participants reported not being employed, three reported working full-time while studying, six reported working part-time while studying, and five reported occasional employment while studying. All data were collected during the 2024/2025 academic year.

The sample size for this study was calculated a priori using G*power software, using a F-test, regarding the linear multiple regression (fixed model, R-squared increase; Faul et al., 2007). Assuming an alpha level of 0.05, a desired power of 0.80, and an effect size of 0.30, the minimum required sample size was 29 participants.

#### 5.1.2. Measure

In addition to the CC-PEC Scale, the following measures were used:

The Satisfaction with Life Scale (SWLS; Diener et al., 1985; Italian validation by Di Fabio & Gori, 2016). Life satisfaction was measured using this 5-item scale, which assesses overall life satisfaction in terms of hedonic well-being. Participants responded on a 7-point Likert scale. Cronbach’s alpha and McDonald’s Omega values were 0.84 and 0.84 at time 1 and 0.88 and 0.88 at time 2, respectively.

The Flourishing Scale (FS; Diener et al., 2010; Italian validation by Giuntoli et al., 2017). Psychological flourishing was measured using this 8-item scale, which assesses eudaimonic well-being. Participants responded on a 7-point Likert scale. Cronbach’s alpha and McDonald’s Omega values were 0.87 and 0.88 at time 1 and 0.91 and 0.91 at time 2, respectively.

### 5.2. Data Analysis

Regression analyses were conducted to evaluate predictive validity, aiming to ascertain the extent to which our measurements could predict life satisfaction and flourishing. Furthermore, test–retest reliability was ensured by calculating intraclass correlation coefficients following a 3-week interval.

### 5.3. Results

Regarding test–retest reliability, the intraclass correlation coefficient for CC-PEC related to COVID-19 pandemic was 0.73 (95% CI [0.47, 0.87]), for CC-PEC related to war was 0.86 (95% CI [0.71, 0.93]), and for CC-PEC related to climate emergency was 0.74 (95% CI [0.49, 0.87]). These findings suggest preliminary evidence of temporal stability; however, given the small sample size, the estimates should be interpreted with caution.

Table 5 shows preliminary associations between the CC-PEC related to war with both life satisfaction and flourishing. For CC-PEC related to climate emergency the regression analysis revealed a negative and significant relationship with flourishing but not with life satisfaction. For CC-PEC related to COVID-19 pandemic, the regression analysis revealed a non-significant relationship with both flourishing and life satisfaction. See Table 7 for more details.

## 6. Discussion

Global crises such as the COVID-19 pandemic, geopolitical conflicts, and the climate emergency are reshaping labour markets and influencing how young people envision their future careers, often increasing uncertainty and complicating career planning. Prior to this study, no instrument was available to assess how these challenges are perceived as barriers to career development. Hence, this study aimed to develop and validate the Career Concerns related to Perceived Environmental Challenges (CC-PEC) Scale through four studies conducted with independent samples of young adults.

From a conceptual perspective, the CC-PEC construct should be distinguished from related psychological constructs such as future anxiety, career distress, and intolerance of uncertainty. While future anxiety refers to a generalized negative anticipation of personal future events (Zaleski et al., 2019), and intolerance of uncertainty reflects a desire for predictability and cognitive paralysis in uncertain situations (Birrell et al., 2011), the CC-PEC scale specifically assesses the interpretation of macro-environmental challenges as career-related barriers. In line with SCCT, the focus is not on emotional reactions per se, but on cognitively mediated perceptions of contextual constraints influencing career planning. Similarly, career-related distress is a common and painful outcome of many negative career experiences (Creed et al., 2016), whereas CC-PEC captures externally perceived environmental threats integrated into career representations. In this sense, the CC-PEC operationalizes the SCCT concept of perceived contextual barriers at a macro-social level, allowing researchers to examine how global environmental instability becomes cognitively integrated into individuals’ career planning processes.

In addition to conceptual distinctions, it is important to position the CC-PEC scale in relation to existing measurement instruments addressing similar global challenges. Existing instruments mainly assess emotional responses to specific global threats (e.g., fear, anxiety, distress), whereas the CC-PEC scale captures how these events are cognitively interpreted as barriers to career development. This distinction helps explain why correlations with fear-based measures were moderate rather than high, supporting the incremental conceptual contribution of the instrument.

The results of Studies 1 and 2 confirmed the appropriateness of the items created to measure career concerns related to COVID-19 pandemic, war, and climate emergency. Specifically, although the three dimensions were assessed using parallel item structures, factor analysis clearly distinguished them according to their environmental referent. This suggests that participants differentiated between the contextual sources of career concerns rather than responding to a generalized form of career anxiety. The findings of Study 2 provide strong support for a hierarchical conceptualization of the construct and for its measurement equivalence across gender. Consistent with the theoretical framework, results supported a three-factor structure (i.e., career-related COVID-19 pandemic concern, career-related war concern, and career-related climate emergency concern) organized under a higher-order general factor, namely Career-related environmental concerns. This hierarchical solution demonstrated satisfactory model fit and outperformed the single-factor model, indicating that career-related environmental concerns are not unidimensional but rather reflect distinguishable yet interrelated domains. The higher-order factor accounted for substantial variance in the first-order dimensions, suggesting a general tendency to perceive environmental challenges as career barriers. At the same time, the adequate loadings of the first-order factors confirm the importance of maintaining domain specificity.

The magnitude of the intercorrelations among the three dimensions warrants a more cautious interpretation of their distinctiveness. The relatively high associations suggest that a substantial portion of the variance may reflect a general tendency to experience environmental challenges as barriers to career development, rather than entirely independent domain-specific concerns. Additional indices (e.g., ECV and ωH) supported a hierarchical structure, indicating that, although a strong general component is present, the multidimensional nature of the construct remains meaningful, with domain-specific factors contributing unique variance. While the second-order model captures shared variance effectively, the proximity between factors suggests potential partial redundancy. This issue may be partly attributable to the parallel item structure, which—while ensuring comparability—could have inflated shared method variance. Overall, these findings support a hierarchical interpretation in which domain-specific concerns coexist with a broader general dimension of career-related environmental concern, while also highlighting the need for future research to further examine the incremental distinctiveness of each dimension, for instance, through the use of non-parallel item formulations and external validation criteria. This dual structure aligns with contemporary perspectives on career development in uncertain contexts, where both general vulnerability to environmental instability and domain-specific threat perceptions coexist.

Using a stepwise approach, full scalar invariance was supported. The establishment of scalar invariance allows for meaningful comparisons of latent means across gender.

Study 3 sought to test the concurrent and discriminant validity of the three dimensions of the CC-PEC scale concerning measures of concerns related to the same three aspects, i.e., the COVID-19 pandemic, war and climate emergency. The correlations, consistent with Studies 1 and 2, were strong between the three dimensions of the CC-PEC scale. On the other hand, the correlations between the sub-dimensions of the scales relating to the same events were weak (e.g., between the CC-PEC related to war and the psychological and experiential fear of war scales, respectively) to moderate (e.g., between the CC-PEC related to the COVID-19 pandemic and the Fear of COVID-19 scale). These findings are consistent with theoretical expectations, as the scales, although related to the same events, measure different constructs. Accordingly, correlations were expected but not necessarily high. The only non-significant relationship concerned career-related war concerns and egoistic environmental concerns. This may reflect the distinct focus of these constructs, as environmental concern has been defined as an “affect associated with beliefs about environmental problems” (Schultz, 2001, p. 31), whereas egoistic concerns are primarily self-oriented and less related to broader contextual threats. Further analyses supported discriminant validity, indicating that the scale captures related but distinct perceptions of these events.

Finally, Study 4 provided preliminary evidence of temporal stability and exploratory indications of predictive validity. The choice of life satisfaction and flourishing as outcome variables was theoretically grounded in the SCCT (Lent et al., 1994, 2000) and LD (Savickas et al., 2009) perspectives, which emphasize the broader impact of contextual barriers on individuals’ overall life adaptation and well-being. Perceived environmental challenges may influence individuals’ general outlook on their general life satisfaction and flourishing, as conceptualized by Diener et al. (2010) as a state characterized by the realization of one’s potential, the presence of positive relationships, and a deep sense of meaning in life. Findings related to war-, climate-, and COVID-related career concerns are broadly consistent with previous research showing that global threats may negatively affect young people’s well-being and future orientation (Moshagen & Hilbig, 2022; Regnoli et al., 2023, 2024a; Riad et al., 2022; Skwirczyńska et al., 2022; Hickman, 2019, 2020; Hickman et al., 2021; Pihkala, 2020; Skilling et al., 2023; Li et al., 2020; Zammitti et al., 2021).

In particular, Study 4 showed that career-related climate emergency concerns and career-related COVID-19 pandemic concerns negatively predicted flourishing. However, contrary to hypotheses, no predictive relationship emerged with life satisfaction. This may be due to the timing of data collection, when concerns related to the COVID-19 pandemic were likely waning and social functioning had partially resumed. These findings should therefore be considered exploratory and hypothesis-generating rather than confirmatory.

In line with SCCT, both objective environmental factors and individuals’ perceptions of them influence career development (Lent et al., 2000). In particular, it is individuals’ perceptions that influence career development by shaping how they interpret and respond to contextual conditions (Vondracek et al., 2019). Identifying and proactively addressing perceived barriers is a key aspect of both SCCT- and LD-based career interventions (Lent, 2013; Lent et al., 2000; Savickas et al., 2009; Savickas & Porfeli, 2012). In this paper, with four studies, the findings indicate that the CC-PEC Scale provides a reliable and valid assessment of career-related concerns associated with major contemporary environmental challenges. It therefore represents a valuable tool for career counselling practice. The CC-PEC scale complements rather than replaces existing fear- or anxiety-based measures, extending assessment toward the career-development domain. From a theoretical perspective, the CC-PEC construct can be further understood within the core mechanisms of SCCT and the LD paradigm. Within SCCT, the perception of environmental challenges as career-related barriers may influence individuals’ self-efficacy beliefs and outcome expectations, thereby shaping goal setting and career-related actions. In particular, perceiving global instability as a constraint may reduce confidence in one’s ability to achieve desired career outcomes or alter expectations regarding future opportunities. Similarly, within the LD framework, these perceived barriers may affect individuals’ capacity for career adaptability, particularly in terms of concern and control, and may influence the way individuals construct and narrate their future career trajectories. Environmental uncertainty may challenge the development of coherent and future-oriented narratives, requiring individuals to engage in more complex meaning-making processes.

The CC-PEC scale therefore represents a valuable tool for both research and practice, complementing existing emotion-based measures by extending assessment to the career-development domain.

Overall, the CC-PEC scale provides a theoretically grounded and empirically validated instrument to assess how macro-contextual uncertainty is cognitively integrated into career development processes, offering important implications for research and career guidance in increasingly uncertain socio-economic contexts.

### Limitations of the Studies and Future Research Directions

A primary limitation concerns the composition of the samples. All four studies relied exclusively on Italian university students, with a marked predominance of female participants. This limits the generalizability of the findings beyond this specific population and raises concerns about their applicability to other age groups, occupational statuses, and cultural contexts. Moreover, the gender imbalance may have influenced the observed levels of environmental career concerns, given known gender differences in risk perception and future-oriented anxieties. Future research should therefore validate the CC-PEC scale in more heterogeneous, gender-balanced samples and across diverse cultural contexts to establish its cross-cultural robustness and broader applicability.

A second limitation concerns the magnitude of the intercorrelations among the three dimensions of the CC-PEC scale. The relatively high associations (ranging from 0.65 to 0.76) suggest that a substantial portion of the variance may reflect a general tendency to experience environmental challenges as barriers to career development, rather than entirely independent domain-specific concerns. Although additional indices (e.g., ECV and ωH) supported a hierarchical structure, indicating the coexistence of general and domain-specific components, these results raise questions about the empirical distinctiveness of the subdimensions. This issue may be partly attributable to the use of parallel item wording across the three domains, which, while ensuring comparability, may have inflated shared method variance and contributed to the observed overlap among dimensions. Future research should therefore develop non-parallel, domain-specific items that capture more distinct aspects of career concerns, in order to more rigorously test the unique contribution of each dimension.

With respect to Study 3, some caution is warranted in interpreting discriminant validity. The Average Variance Extracted (AVE) of the Fear of War Scale (FOWARS) did not reach the recommended threshold, which may limit the strict application of the Fornell–Larcker criterion. As a result, shared variance estimates involving this construct may be inflated. Nevertheless, this limitation concerns the external measure rather than the CC-PEC scale itself. Importantly, the overall pattern of correlations remains theoretically coherent, supporting the distinction between career-related concerns and affective fear-based responses. Future studies should include external variables that are more clearly differentiated to provide a more stringent test of discriminant validity.

More broadly, further research is needed to examine the predictive role of CC-PEC within the SCCT framework. In line with SCCT, perceived barriers influence both career goals and actions and may moderate the relationships between interests, choices, and outcomes. Future studies should therefore investigate whether the constructs measured by the CC-PEC scale operate within these mechanisms, extending the examination of predictive validity beyond well-being indicators such as flourishing and life satisfaction.

A further limitation pertains to Study 4, which was based on a small and gender-okimbalanced sample (*n* = 34). Although consistent with minimum requirements derived from a priori power analysis, this sample size does not allow robust inferences regarding predictive validity. Estimates derived from small samples are inherently unstable and associated with reduced precision, limiting confidence in the observed effects. Accordingly, these findings should be regarded as preliminary and require replication in larger samples to establish the stability and reliability of the reported relationships.

In conclusion, it is important to stress the limitation of the use of parallel item wording across the three environmental challenges. Although this approach ensured strict comparability across contexts, it may have contributed to the relatively high intercorrelations among the dimensions. Future research could address this issue by developing event-specific items that capture qualitatively distinct aspects of career concerns beyond shared economic and employment-related worries.

## 7. Conclusions

This study developed and validated, across four studies, a novel instrument to measure career concerns related to perceived environmental challenges, the CC-PEC Scale. The environmental challenges considered are the COVID-19 pandemic, war and climate emergency. Beyond its psychometric contribution, the CC-PEC Scale addresses a relevant gap within the Social Cognitive Career Theory (SCCT) and Life Design (LD) paradigms. While both frameworks emphasize the role of perceived contextual barriers in shaping career development, no instrument was previously available to assess how large-scale environmental threats are internalized as career-related concerns. The CC-PEC Scale operationalizes these macro-contextual barriers, allowing empirical investigation of how global instability is cognitively integrated into career planning processes. One of the studies provided exploratory evidence suggesting that career concerns related to war may negatively affect both life satisfaction and flourishing, and that career concerns related to climate emergency might negatively affect flourishing. Hence, it might be particularly important to consider these aspects. We believe that the use of the CC-PEC scale could be effectively applied to the practice of career counsellors, allowing them to reveal problematic environmental barrier perceptions that could hinder career development and to help clients overcome them. The CC-PEC scale will also help researchers explore the impact of current environmental challenges on career development and their relationships with adaptability resources and career and well-being outcomes. Furthermore, the scale offers researchers a tool to investigate how contemporary environmental instability interacts with adaptability, self-efficacy, and career and well-being outcomes within SCCT and LD frameworks.

## Figures and Tables

**Table 1:** Descriptive statistics, commonalities and factor loadings.

Item	M (SD)	Skewness	Kurtosis	Commonalities	Factor 1	Factor 2	Factor 3
1. I worry about future employment because of a potential economic recession due to the outbreak of COVID-19.	2.91 (1.04)	−0.06	−0.61	0.48	0.70		
2. I worry about future employment because of fierce competition in the job market due to the outbreak of COVID-19.	2.92 (1.08)	−0.03	−0.72	0.59	0.84		
3. I worry about future employment because my salary would probably not be as excellent as I wish for the devastating effect of COVID-19.	2.77 (1.08)	0.13	−0.74	0.53	0.69		
4. I worry about future employment because of the increasing unemployment and job cut reported by the mass media for the reason of COVID-19.	3.19 (1.07)	−0.19	−0.66	0.57	0.73		
5. I worry about future employment because I probably would not find a job that interests me for the reason of COVID-19.	2.29 (1.06)	0.52	−0.44	0.47	0.44		
6. I worry about future employment because of a potential economic recession due to the war in Ukraine.	3.14 (1.15)	−0.01	−0.89	0.54		0.80	
7. I worry about future employment because of fierce competition in the job market due to the war in Ukraine.	2.61 (1.10)	0.24	−0.72	0.65		0.81	
8. I worry about future employment because my salary would probably not be as excellent as I wish for the devastating effect of the war in Ukraine.	2.69 (1.16)	0.26	−0.78	0.74		0.90	
9. I worry about future employment because of the increasing unemployment and job cut reported by the mass media for the war in Ukraine.	2.57 (1.09)	0.37	−0.46	0.66		0.67	
10. I worry about future employment because I probably would not find a job that interests me for the war in Ukraine.	2.10 (1.01)	0.57	−0.47	0.62		0.60	
11. I worry about future employment because of a potential economic recession due to the climate emergency.	3.04 (1.20)	−0.01	−0.99	0.49			0.68
12. I worry about future employment because of fierce competition in the job market due to the climate emergency.	2.65 (1.09)	0.24	−0.58	0.74			0.89
13. I worry about future employment because my salary would probably not be as excellent as I wish for the devastating effect of climate emergency.	2.58 (1.14)	0.35	−0.59	0.79			0.94
14. I worry about future employment because of the increasing unemployment and job cut reported by the mass media for the reason of climate emergency.	2.62 (1.17)	0.32	−0.80	0.72			0.86
15. I worry about future employment because I probably would not find a job that interests me for the reason of to climate emergency.	2.47 (1.17)	0.46	−0.64	0.61			0.69

**Table 2:** Goodness-of-fit statistics for competing CFA models.

Model	χ^2^	df	RMSEA	CFI	AIC	BIC
Single-factor	818.40	90	0.189	0.608	9035.03	9188.95
First-order (correlated)	212.62	87	0.080	0.932	8265.29	8429.48
Second-order (hierarchical)	214.01	88	0.080	0.932	8264.55	8425.31
Bifactor (hierarchical)	198.32	75	0.085	0.934	8257.83	8463.07

**Note.** χ^2^ = chi-square statistic; df = degrees of freedom; RMSEA = root mean square error of approximation; CFI = comparative fit index; AIC = Akaike information criterion; BIC = Bayesian information criterion. Lower AIC and BIC values indicate better model fit.

**Table 3:** Chi-square difference tests and incremental fit changes.

Comparison	Δχ^2^	Δdf	*p*	|ΔCFI|	|ΔRMSEA|
Single-factor vs. First-order	605.78	3	<0.001	0.324	0.109
Second-order vs. First-order	1.40	1	0.237	0.000	0.000
Bifactor vs. First-order	14.30	12	0.282	0.002	0.005

**Note.** Model comparisons were evaluated using Δχ^2^ (DIFFTEST), ΔCFI, and ΔRMSEA. A model was considered worse if at least two of the following criteria were exceeded: *p* ≤ 0.050 for Δχ^2^, ΔCFI ≥ 0.01, and ΔRMSEA ≥ 0.015 (Chen, 2007).

**Table 4:** Measurement invariance across gender.

Model	χ^2^	df	CFI	RMSEA	SRMR	Δχ^2^	Δdf	*p*	|ΔCFI|	|ΔRMSEA|
Configural	361.29	174	0.906	0.098	0.062	—	—	—	—	—
Metric	377.22	186	0.904	0.095	0.067	12.89	12	0.377	0.002	0.003
Scalar	387.55	198	0.905	0.092	0.069	8.38	12	0.755	0.001	0.003

**Note.** Δχ^2^ tests refer to the comparison with the less constrained model (Metric vs. Configural; Scalar vs. Metric). Fit indices are from MLR estimation. RMSEA = root mean square error of approximation; CFI = comparative fit index.

**Table 5:** Pearson’s correlation.

Variables	M (SD)	1.	2.	3.	4.	5.	6.	7.	8.
1. Career-related COVID-19 pandemic concern	2.71 (0.99)	-							
2. Career-related war concern	2.74 (1.03)	0.76 **	-						
3. Career-related climate emergency concern	2.68 (1.08)	0.65 **	0.66 **	-					
4. Physiological Fear of War	4.04 (0.77)	0.19 **	0.19 **	0.16 **	-				
5. Experiential Fear of War	2.80 (1.03)	0.24 **	0.27 **	0.22 **	0.45 **	-			
6. Fear of COVID-19	2.59 (1.17)	0.39 **	0.29 **	0.26 **	0.31 **	0.32 **	-		
7. Environmental biospheric concerns	5.67 (1.27)	0.09	0.10	0.19 **	0.37 **	0.16 **	0.12 *	-	
8. Environmental egoistic concerns	5.72 (1.13)	0.09	0.13 *	0.09	0.39 **	0.11	0.14 *	0.36 **	-

**Note.** * *p* < 0.05. ** *p* < 0.01.

**Table 6:** Average Variance Extracted (AVE) and squared correlations.

Variables	AVE	R^2^							
		1.	2.	3.	4.	5.	6.	7.	8.
1. Career-related COVID-19 pandemic concern	0.64	-							
2. Career-related war concern	0.66	0.58	-						
3. Career-related climate emergency concern	0.73	0.42	0.43	-					
4. Physiological Fear of War	0.46	0.04	0.04	0.02	-				
5. Experiential Fear of War	0.52	0.06	0.07	0.05	0.20	-			
6. Fear of COVID-19	0.76	0.15	0.08	0.07	0.10	0.10	-		
7. Environmental biospheric concerns	0.76	0.01	0.01	0.04	0.14	0.02	0.01	-	
8. Environmental egoistic concerns	0.61	0.01	0.02	0.01	0.15	0.01	0.02	0.13	-

**Note.** AVE = Average Variance Extracted, R^2^ = squared correlations.

**Table 7:** Results of the predictive validity of career-related concern scale on life satisfaction and flourishing.

Dependent Variable	Predictor	β	t	*p*	Model Statistics
Life satisfaction	Career-related COVID-19 pandemic concern	−0.50	−0.28	0.78	R^2^ = 0.002
					F = 0.08
Flourishing	Career-related COVID-19 pandemic concern	−0.29	−1.72	0.09	R^2^ = 0.08
					F = 2.97
Life satisfaction	Career-related war concern	−0.38	−2.33	0.03	R^2^ = 0.14
					F = 5.44
Flourishing	Career-related war concern	−0.58	−4.04	0.000	R^2^ = 0.34
					F = 16.35
Life satisfaction	Career-related climate emergency concern	−0.25	−1.49	0.14	R^2^ = 0.06
					F = 2.23
Flourishing	Career-related climate emergency concern	−0.47	−2.99	0.005	R^2^ = 0.22
					F = 8.91

## Data Availability

The data presented in this study are available on request from the corresponding author due to privacy reasons.

## Abbreviations

The following abbreviations are used in this manuscript:

CC-PEC	Career Concerns related to Perceived Environmental Challenges
SCCT	Social Cognitive Career Theory
LD	Life Design
ILO	International Labour Organisation
EFA	Exploratory Factor Analysis
KMO	Kaiser–Meyer–Olkin
M	Mean
SD	Standard Deviation
CFA	Confirmatory Factor Analysis
SB χ^2^	Satorra–Bentler Chi-square test
CFI	Comparative Fit Index
TLI	Tucker–Lewis index
RMSEA	Root Mean Square Error of Approximation
SRMR	Standardized Root Mean Square Residual
AIC	Akaike Information Criterion
AVE	Average Variance Extracted

## References

[B1-behavsci-16-00636] Ahorsu D. K., Lin C. Y., Imani V., Saffari M., Griffiths M. D., Pakpour A. H. (2020). The fear of COVID-19 scale: Development and initial validation. International Journal of Mental Health and Addiction.

[B2-behavsci-16-00636] Andresen S., Heyer L., Lips A., Rusack T., Thomas S., Schröer W., Thomas S., Wilmes J. (2021). Das Leben von jungen Menschen in der Corona-Pandemie. Erfahrungen, Sorgen, Bedarfe.

[B3-behavsci-16-00636] Bandura A. (1986). Social foundations of thought and action.

[B4-behavsci-16-00636] Barchielli B., Cricenti C., Gallè F., Sabella E. A., Liguori F., Da Molin G., Liguori G., Orsi G. B., Giannini A. M., Ferracuti S., Napoli C. (2022). Climate changes, natural resources depletion, COVID-19 pandemic, and Russian-Ukrainian war: What is the impact on habits change and mental health?. International Journal of Environmental Research and Public Health.

[B5-behavsci-16-00636] Beaton D. E., Bombardier C., Guillemin F., Ferraz M. B. (2000). Guidelines for the process of cross-cultural adaptation of self-report measures. Spine.

[B6-behavsci-16-00636] Bentler P. M. (1990). Comparative fit indexes in structural models. Psychological Bulletin.

[B7-behavsci-16-00636] Bentler P. M. (1998). EQS structural equations program manual.

[B8-behavsci-16-00636] Birrell J., Meares K., Wilkinson A., Freeston M. (2011). Toward a definition of intolerance of uncertainty: A review of factor analytical studies of the intolerance of uncertainty scale. Clinical Psychology Review.

[B9-behavsci-16-00636] Browne M. W., Cudeck R. (1992). Alternative ways of assessing model fit. Sociological Methods & Research.

[B10-behavsci-16-00636] Burnham K. P., Anderson D. R. (2004). Multimodel inference: Understanding AIC and BIC in model selection. Sociological Methods & Research.

[B11-behavsci-16-00636] Cebeci F., Reyes M. E. S., Innocenti M., Kochuchakkalackal G., Jeremie W., Buvar A., Atak I., Karaman M., Dinçer R., Cankardaş Nalbantçılar S., Mammadov E., Cadeddu C., Pacquing M. C. T., Ágoston C., Santarelli G., Cayubit R. F. O., Kuttiankal T., Uzun K., Trinidad K. K. V., Artan T. (2025). Eco-anxiety without borders: A cross-national study on climate perceptions, beliefs about government climate action, and climate concern. International Journal of Social Psychiatry.

[B12-behavsci-16-00636] Chen F. F. (2007). Sensitivity of goodness of fit indexes to lack of measurement invariance. Structural Equation Modeling.

[B13-behavsci-16-00636] Child D. (2006). The essentials of factor analysis.

[B15-behavsci-16-00636] Ciobanu G., Dinu M., Iacob O. C., Constantinescu V. G. (2022). Digital labour market model and financial opportunities in the context of sustainable development in the EU countries. European Journal of Sustainable Development.

[B14-behavsci-16-00636] Clark L. A., Watson D. B. (1995). Constructing validity: Basic issues in objective scale development. Psychological Assessment.

[B16-behavsci-16-00636] Costello A. B., Osborne J. (2005). Best practices in exploratory factor analysis: Four recommendations for getting the most from your analysis. Practical Assessment, Research, and Evaluation.

[B17-behavsci-16-00636] Creed P. A., Hood M., Praskova A., Makransky G. (2016). The career distress scale: Using Rasch measurement theory to evaluate a brief measure of career distress. Journal of Career Assessment.

[B18-behavsci-16-00636] Crocker L. M., Algina J. (1986). Introduction to classical and modern test theory.

[B19-behavsci-16-00636] Diener E., Emmons R. A., Larsen R. J., Griffin S. (1985). The satisfaction with life scale. Journal of Personality Assessment.

[B20-behavsci-16-00636] Diener E., Wirtz D., Tov W., Kim-Prieto C., Choi D. W., Oishi S., Biswas-Diener R. (2010). New well-being measures: Short scales to assess flourishing and positive and negative feelings. Social Indicators Research.

[B21-behavsci-16-00636] Di Fabio A., Gori A. (2016). Measuring adolescent life satisfaction: Psychometric properties of the satisfaction with life scale in a sample of Italian adolescents and young adults. Journal of Psychoeducational Assessment.

[B22-behavsci-16-00636] Di Maggio I., Ginevra M. C., Santilli S., Nota L., Soresi S. (2020). The role of career adaptability, the tendency to consider systemic challenges to attain a sustainable development, and hope to improve investments in higher education. Frontiers in Psychology.

[B23-behavsci-16-00636] Duarte M. E. (2018). Life design paradigm: A perspective and practice for career counseling in the twenty-first century. Diversity in harmony–insights from psychology: Proceedings of the 31st international congress of psychology.

[B25-behavsci-16-00636] Farrell A. M. (2010). Insufficient discriminant validity: A comment on Bove, Pervan, Beatty, and Shiu (2009). Journal of Business Research.

[B26-behavsci-16-00636] Faul F., Erdfelder E., Lang A. G., Buchner A. (2007). G* Power 3: A flexible statistical power analysis program for the social, behavioral, and biomedical sciences. Behavior Research Methods.

[B24-behavsci-16-00636] Field A. (2013). Discovering statistics using SPSS.

[B27-behavsci-16-00636] Fornell C., Larcker D. F. (1981). Evaluating structural equation models with unobservable variables and measurement error. Journal of Marketing Research.

[B28-behavsci-16-00636] George D., Mallery P. (2010). SPSS for Windows step by step: A simple guide and reference.

[B29-behavsci-16-00636] Ginevra M. C., Di Maggio I., Santilli S., Nota L. (2021). Italian adolescents’ understandings of globalization. Journal of Adolescence.

[B30-behavsci-16-00636] Giuntoli L., Ceccarini F., Sica C., Caudek C. (2017). Validation of the Italian versions of the flourishing scale and of the scale of positive and negative experience. Sage Open.

[B31-behavsci-16-00636] Grieder S., Steiner M. D. (2021). Algorithmic jingle jungle: A comparison of implementations of principal axis factoring and promax rotation in R and SPSS. Behavior Research Methods.

[B32-behavsci-16-00636] Hair J. F., Anderson R. E., Tatham R. L., Black W. C. (1998). Multivariate data analysis.

[B33-behavsci-16-00636] Hair J. F., Black W. C., Babin B. J., Anderson R. E. (2010). Multivariate data analysis.

[B34-behavsci-16-00636] Hickman C., Hoggett P. (2019). Children and climate change: Exploring children’s feelings about climate change using free association narrative interview methodology. Climate psychology. Studies in the psychosocial.

[B35-behavsci-16-00636] Hickman C. (2020). We need to (find a way to) talk about… Eco-anxiety. Journal of Social Work Practice.

[B36-behavsci-16-00636] Hickman C., Marks E., Pihkala P., Clayton S., Lewandowski R. E., Mayall E. E., Wray B., Mellor C., van Susteren L. (2021). Climate anxiety in children and young people and their beliefs about government responses to climate change: A global survey. The Lancet Planetary Health.

[B37-behavsci-16-00636] Hogg T. L., Stanley S. K., O’Brien L. V., Wilson M. S., Watsford C. R. (2021). The Hogg eco-anxiety scale: Development and validation of a multidimensional scale. Global Environmental Change.

[B38-behavsci-16-00636] Horn J. L. (1965). A rationale and test for the number of factors in factor analysis. Psychometrika.

[B39-behavsci-16-00636] Hu L. T., Bentler P. M. (1999). Cutoff criteria for fit indexes in covariance structure analysis: Conventional criteria versus new alternatives. Structural Equation Modeling: A Multidisciplinary Journal.

[B40-behavsci-16-00636] Innocenti M. (2022). Ecoansia: I cambiamenti climatici tra attivismo e paura.

[B41-behavsci-16-00636] Innocenti M., Comerci C., Dockerty G., Grassi G., Santarelli G., Cadeddu C. (2025). From eco-anxiety to eco-paralysis: A case study on behavioral responses to climate change in healthcare professionals. The Journal of Climate Change and Health.

[B42-behavsci-16-00636] International Labor Organization (2020). Young workers will be hit hard by COVID-19’s economic fallout.

[B43-behavsci-16-00636] Iovu M. B., Hărăguș P. T., Roth M. (2018). Constructing future expectations in adolescence: Relation to individual characteristics and ecological assets in family and friends. International Journal of Adolescence and Youth.

[B44-behavsci-16-00636] Kaiser H. F., Rice J. (1974). Little jiffy, mark IV. Educational and Psychological Measurement.

[B45-behavsci-16-00636] Kalcza-Janosi K., Kotta I., Marschalko E. E., Szabo K. (2023). The fear of war scale (FOWARS): Development and initial validation. Social Sciences.

[B46-behavsci-16-00636] Korkmaz O., Doğanülkü H. A. (2021). Fear of COVID-19 and career distress: Mediating role of visions about the future. İş ve İnsan Dergisi.

[B47-behavsci-16-00636] Lent R. W. (2013). Career-life preparedness: Revisiting career planning and adjustment in the new workplace. The Career Development Quarterly.

[B48-behavsci-16-00636] Lent R. W., Brown S. D., Hackett G. (1994). Toward a unifying social cognitive theory of career and academic interest, choice, and performance. Journal of Vocational Behavior.

[B49-behavsci-16-00636] Lent R. W., Brown S. D., Hackett G. (2000). Contextual supports and barriers to career choice: A social cognitive analysis. Journal of Counseling Psychology.

[B50-behavsci-16-00636] Lewchuk W. (2017). Precarious jobs: Where are they, and how do they affect well-being?. The Economic and Labour Relations Review.

[B51-behavsci-16-00636] Li P., Fu J. B., Li K. F., Liu J. N., Wang H. L., Liu L. J., Chen Y., Zhang Y. L., Liu S. L., Tang A., Tong Z. D., Yan J. B. (2020). Transmission of COVID-19 in the terminal stages of the incubation period: A familial cluster. International Journal of Infectious Diseases.

[B52-behavsci-16-00636] Mahmud M. S., Talukder M. U., Rahman S. M. (2021). Does ‘Fear of COVID-19’ trigger future career anxiety? An empirical investigation considering depression from COVID-19 as a mediator. International Journal of Social Psychiatry.

[B53-behavsci-16-00636] Margolius M., Doyle Lynch A., Pufall Jones E., Hynes M. (2020). The state of young people during COVID-19: Findings from a nationally representative survey of high school youth.

[B54-behavsci-16-00636] Moshagen M., Hilbig B. E. (2022). Citizens’ psychological reactions following the Russian invasion of Ukraine: A cross-national study [Preprint]. PsyArXiv.

[B55-behavsci-16-00636] Nota L., Soresi S., Ferrari L., Ginevra M. C. (2014). Vocational designing and career counseling in Europe. European Psychologist.

[B56-behavsci-16-00636] Orhan E. (2022). The effects of the Russia-Ukraine war on global trade. Journal of International Trade, Logistics and Law.

[B57-behavsci-16-00636] Osborne J. W., Costello A. B., Kellow J. T., Osborne J. W. (2008). Best practices in exploratory factor analysis. Best practices in quantitative methods.

[B58-behavsci-16-00636] Parola A. (2020). Novel coronavirus outbreak and career development: A narrative approach into the meaning for Italian university graduates. Frontiers in Psychology.

[B59-behavsci-16-00636] Pihkala P. (2020). Anxiety and the ecological crisis: An analysis of eco-anxiety and climate anxiety. Sustainability.

[B60-behavsci-16-00636] Polit D. F., Beck C. T. (2020). Essentials of nursing research: Appraising evidence for nursing practice.

[B61-behavsci-16-00636] Regnoli G. M., Tiano G., De Rosa B. (2023). Italian Adaptation and validation of the fear of war scale and the impact of the fear of war on young Italian adults’ mental health. Social Sciences.

[B62-behavsci-16-00636] Regnoli G. M., Tiano G., De Rosa B. (2024a). How is the fear of war impacting Italian young adults’ mental health? The mediating role of future anxiety and intolerance of uncertainty. European Journal of Investigation in Health, Psychology and Education.

[B63-behavsci-16-00636] Regnoli G. M., Tiano G., De Rosa B. (2024b). Serial mediation models of future anxiety and Italian young adults psychological distress: The role of intolerance of uncertainty and non-pathological worry. European Journal of Investigation in Health, Psychology and Education.

[B64-behavsci-16-00636] Riad A., Drobov A., Krobot M., Antalová N., Alkasaby M. A., Peřina A., Koščík M. (2022). Mental health burden of the Russian–Ukrainian war 2022 (RUW-22): Anxiety and depression levels among young adults in central Europe. International Journal of Environmental Research and Public Health.

[B65-behavsci-16-00636] Rifkin J. (2019). The green new deal: Why the fossil fuel civilization will collapse by 2028, and the bold economic plan to save life on earth.

[B66-behavsci-16-00636] Rocchi G., Pileri J., Luciani F., Gennaro A., Lai C. (2023). Insights into eco-anxiety in Italy: Preliminary psychometric properties of the Italian version of the Hogg eco-anxiety scale, age and gender distribution. Journal of Environmental Psychology.

[B67-behavsci-16-00636] Rodriguez A., Reise S. P., Haviland M. G. (2016). Evaluating bifactor models: Calculating and interpreting statistical indices. Psychological Methods.

[B68-behavsci-16-00636] Santilli S., Ginevra M. C., Di Maggio I., Soresi S., Nota L. (2021). In the same boat? An online group career counseling with a group of young adults in the time of COVID-19. International Journal for Educational and Vocational Guidance.

[B69-behavsci-16-00636] Santilli S., Ginevra M. C., Di Maggio I., Soresi S., Nota L. (2023). Construction and initial validation of the scale “goals for future design of the 2030 agenda”. International Journal for Educational and Vocational Guidance.

[B70-behavsci-16-00636] Satorra A., Bentler P. M. (2001). A scaled difference chi-square test statistic for moment structure analysis. Psychometrika.

[B71-behavsci-16-00636] Savickas M. L. (1997). Career adaptability: An integrative construct for life-span, life-space theory. The Career Development Quarterly.

[B72-behavsci-16-00636] Savickas M. L., Nota L., Rossier J., Dauwalder J. P., Duarte M. E., Guichard J., Soresi S., Van Esbroeck R., Van Vianen A. E. (2009). Life designing: A paradigm for career construction in the 21st century. Journal of Vocational Behavior.

[B73-behavsci-16-00636] Savickas M. L., Porfeli E. J. (2012). Career adapt-abilities scale: Construction, reliability, and measurement equivalence across 13 countries. Journal of Vocational Behavior.

[B74-behavsci-16-00636] Schultz P. W. (2001). The structure of environmental concern: Concern for self, other people, and the biosphere. Journal of Environmental Psychology.

[B75-behavsci-16-00636] Sheather J. (2022). As Russian troops cross into Ukraine, we need to remind ourselves of the impact of war on health. BMJ.

[B76-behavsci-16-00636] Shepherd N. (2023). Rethinking heritage in precarious times: Coloniality, climate change, and COVID-19.

[B77-behavsci-16-00636] Skilling P., Hurd F., Lips-Wiersma M., McGhee P. (2023). Navigating hope and despair in sustainability education: A reflexive roadmap for being with eco-anxiety in the classroom. Management Learning.

[B78-behavsci-16-00636] Skwirczyńska E., Kozłowski M., Nowak K., Wróblewski O., Sompolska-Rzechuła A., Kwiatkowski S., Cymbaluk-Płoska A. (2022). Anxiety assessment in Polish students during the Russian–Ukrainian war. International Journal of Environmental Research and Public Health.

[B79-behavsci-16-00636] Soraci P., Ferrari A., Abbiati F. A., Del Fante E., De Pace R., Urso A., Griffiths M. D. (2020). Validation and psychometric evaluation of the Italian version of the fear of COVID-19 scale. International Journal of Mental Health and Addiction.

[B80-behavsci-16-00636] Stebleton M. J. (2010). Narrative-based career counseling perspectives in times of change: An analysis of strengths and limitations. Journal of Employment Counseling.

[B81-behavsci-16-00636] Steiger J. H. (1990). Structural model evaluation and modification: An interval estimation approach. Multivariate Behavioral Research.

[B82-behavsci-16-00636] Tabachnick B. G., Fidell L. S. (2019). Using multivariate statistics.

[B83-behavsci-16-00636] Vondracek F. W., Lerner R. M., Schulenberg J. E. (2019). Career development: A life-span developmental approach.

[B84-behavsci-16-00636] Weigel R., Weigel J. (1978). Environmental concern: The development of a measure. Environment and Behavior.

[B85-behavsci-16-00636] Wheaton B., Muthen B., Alwin D. F., Summers G. F. (1977). Assessing reliability and stability in panel models. Sociological Methodology.

[B86-behavsci-16-00636] World Health Organization (2022). WHO’s response to the Ukraine crisis: Annual report, 2022.

[B87-behavsci-16-00636] Wu J., Snell G., Samji H. (2020). Climate anxiety in young people: A call to action. The Lancet Planetary Health.

[B88-behavsci-16-00636] Wu P., Fang Y., Guan Z., Fan B., Kong J., Yao Z., Liu X., Fuller C. J., Susser E., Lu J. (2009). The psychological impact of the SARS epidemic on hospital employees in China: Exposure, risk perception, and altruistic acceptance of risk. Canadian Journal of Psychiatry.

[B89-behavsci-16-00636] Zaleski Z., Sobol-Kwapinska M., Przepiorka A., Meisner M. (2019). Development and validation of the dark future scale. Time & Society.

[B90-behavsci-16-00636] Zammitti A., Imbrogliera C., Russo A., Zarbo R., Magnano P. (2021). The psychological impact of Coronavirus pandemic restrictions in Italy. The mediating role of the fear of COVID-19 in the relationship between positive and negative affect with positive and negative outcomes. European Journal of Investigation in Health, Psychology and Education.

[B91-behavsci-16-00636] Zammitti A., Russo A., Ginevra M. C., Magnano P. (2023a). “Imagine your career after the COVID-19 pandemic”: An online group career counseling training for university students. Behavioral Sciences.

[B92-behavsci-16-00636] Zammitti A., Santisi G., Magnano P., Di Nuovo S. (2023b). Analyzing attitudes to promote sustainability: The adaptation of the environmental concern scale (ECs) to the Italian context. Sustainability.

